# One-year risk of psychiatric hospitalization and associated treatment costs in bipolar disorder treated with atypical antipsychotics: a retrospective claims database analysis

**DOI:** 10.1186/1471-244X-11-6

**Published:** 2011-01-07

**Authors:** Edward Kim, Min You, Andrei Pikalov, Quynh Van-Tran, Yonghua Jing

**Affiliations:** 1 Bristol-Myers Squibb, Plainsboro, NJ, USA; 2 Otsuka America Pharmaceutical, Inc., Rockville, MD, USA

## Abstract

**Background:**

This study compared 1-year risk of psychiatric hospitalization and treatment costs in commercially insured patients with bipolar disorder, treated with aripiprazole, ziprasidone, olanzapine, quetiapine or risperidone.

**Methods:**

This was a retrospective propensity score-matched cohort study using the Ingenix Lab/Rx integrated insurance claims dataset. Patients with bipolar disorder and 180 days of pre-index enrollment without antipsychotic exposure who received atypical antipsychotic agents were followed for up to 12 months following the initial antipsychotic prescription. The primary analysis used Cox proportional hazards regression to evaluate time-dependent risk of hospitalization, adjusting for age, sex and pre-index hospitalization. Generalized gamma regression compared post-index costs between treatment groups.

**Results:**

Compared to aripiprazole, ziprasidone, olanzapine and quetiapine had higher risks for hospitalization (hazard ratio 1.96, 1.55 and 1.56, respectively; p < 0.05); risperidone had a numerically higher but not statistically different risk (hazard ratio 1.37; p = 0.10). Mental health treatment costs were significantly lower for aripiprazole compared with ziprasidone (p = 0.004) and quetiapine (p = 0.007), but not compared to olanzapine (p = 0.29) or risperidone (p = 0.80). Total healthcare costs were significantly lower for aripiprazole compared to quetiapine (p = 0.040) but not other comparators.

**Conclusions:**

In commercially insured adults with bipolar disorder followed for 1 year after initiation of atypical antipsychotics, treatment with aripiprazole was associated with a lower risk of psychiatric hospitalization than ziprasidone, quetiapine, olanzapine and risperidone, although this did not reach significance with the latter. Aripiprazole was also associated with significantly lower total healthcare costs than quetiapine, but not the other comparators.

## Background

Bipolar disorder is a chronic, recurring disorder associated with periodic disruptions in mood regulation, with annual treatment costs of $7,200 to $12,100 per year, 20% of which are attributable to hospitalizations [1,2]. Acute mania may require hospitalization for stabilization of behavioral dyscontrol, irritability, and risk-taking behavior. Despite the availability of multiple approved medication therapies, more than 75% of patients with bipolar disorder report at least one lifetime psychiatric hospitalization [3].

Medication treatment patterns are variable in the acute and long-term management of bipolar disorder, with 42-64% of patients receiving mood stabilizers, such as lithium, valproate or carbamazapine, and 44-60% receiving adjunctive antipsychotics [4-6]. Atypical antipsychotics are used alone or in combination with mood stabilizers for more severe manic episodes [7-11]. Moreover, adjunctive mood stabilizer-atypical antipsychotic combination treatments may help to prevent psychiatric hospitalization in bipolar disorder [12].

In a recent commercial claims database study, adjunctive aripiprazole was found to be associated with a longer time to initial psychiatric hospitalization than ziprasidone, olanzapine, quetiapine and risperidone during the first 90 days following initiation [13]. A subsequent analysis found that total healthcare expenditures were lower for aripiprazole than ziprasidone, olanzapine and risperidone, and mental health expenditures were lower for aripiprazole than all comparators [14].

The objective of the current study was to assess the 1-year risk of psychiatric hospitalization and associated treatment costs in commercially insured patients with bipolar disorder newly treated with aripiprazole, ziprasidone, olanzapine, quetiapine or risperidone, alone or in combination with mood stabilizers.

## Methods

### Study design

The study was a retrospective cohort study utilizing the Ingenix I3/LabRx claims dataset from 1/1/2003 through 12/31/2006. The dataset is a proprietary sample of individuals receiving health insurance benefits from United Health Care (UHC). UHC data include the inpatient, outpatient and prescription drug claims of more than 15 million of covered lives across the United States. The index date was the date of the first prescription claim for an atypical antipsychotic. Patients were followed for up to 1 year post-index. Because the dataset in this study was derived from an insurance claim database and the data conform to the Health Insurance Portability and Accountability Act of 1996 confidentiality requirements, the study did not require informed consent or institutional review board approval.

### Inclusion criteria

The study included outpatients aged 18-65 years with an ICD-9 code for bipolar disorder, manic, mixed or hypomanic (296.0x, 296.1, 296.4x, 6x, 7x, 8x). Eligible patients required at least 180 days or continuous health plan enrollment before, and 365 days after, the index date. Patients were included only if they were treated on a single atypical antipsychotic at index.

### Exclusion criteria

Patients were excluded from the analysis if they resided in a nursing home, hospice, or another type of long-term care facility, received mail-order prescriptions, or were diagnosed with a schizophrenia spectrum disorder (295.xx) during the pre- or post-index study period. Patients were also excluded if they used any atypical antipsychotic in the 180-day pre-index period, or had prescriptions for more than one atypical antipsychotic at index. Additionally, patients were also excluded if they were hospitalized within 7 days of their index antipsychotic prescription, in order to reduce treatment selection bias based on extreme agitation or instability.

### Assessments and statistical analyses

The primary outcome of interest was the first psychiatric hospitalization in the follow-up period. Patients were censored for the following events: medical hospitalization, discontinuation of index antipsychotic (>15 days gap in coverage), or a prescription for a different antipsychotic during the follow-up period.

In order to control for treatment selection bias, we employed propensity score matching to construct comparison groups that shared similar demographic and clinical characteristics. Propensity score matching is a robust means of controlling for observed confounding in observational data [15]. Propensity scores were calculated for each patient using logistic regression with independent variables of age, sex, region, pre-index diagnosis or treatment of diabetes or hyperlipidemia, pre-index psychiatric hospitalization, pre-index lipid or glucose laboratory claims, choice of pre-index mood stabilizer exposure and Charlson comorbidity index. The propensity score was the predicted probability of treatment calculated for each patient in the regression model. Patients in comparison treatment groups were matched 1:1 if their propensity scores were within 0.25 standard deviations of the logit of the propensity score. All analyses were conducted in propensity score-matched cohorts of the study sample.

The primary analysis used Cox proportional hazards regression to assess time-dependent risk of post-index psychiatric hospitalization with a pre-specified threshold for statistical significance of p < 0.05. Covariates for adjustment in the models included age, sex, diagnosis or treatment for diabetes or hyperlipidemia diagnosis, pre-index psychiatric hospitalization, pre-index lipid or glucose laboratory claims, choice of pre-index mood stabilizer and the Deyo Charlson comorbidity index [16]. Intent-to-treat analysis was used for the cost analysis. Monthly treatment costs during the follow-up period were compared using generalized gamma regression controlling for pre-index costs in patients with positive post-index healthcare costs. First, we calculated the mean for each of the numeric covariates, and gave equal share of the categorical covariates, and then calculated the log mean of the fitted gamma distribution based on these covariate values and the parameter estimates and then exponentiated the log mean to get the cost in dollars. Gamma regressions were used to compare outcomes because gamma distribution is suggested by many as a close approximation of cost data. For example, Diehr and colleagues compared different methods to model healthcare cost data and concluded that, for understanding the effect of individual covariates on total costs, the gamma distribution might be preferred because it is a multiplicative model [17]. Generalized gamma regression has been found to be a more robust estimator than traditional ordinary least squares regression in the analysis of healthcare expenditure data due to the distributional qualities of healthcare costs [18]. Only patients with positive healthcare costs in the follow-up period were included in the analysis, which categorized costs into mental health (inpatient/ER and outpatient), medical (inpatient/ER and outpatient) and pharmacy (all medications used). We excluded patients with non-positive costs based on the assumption that patients taking medications were also receiving billable services and that the absence of such costs reflected aberrant data.

As a sensitivity analysis, we also replicated all multivariate regression analyses on the full unmatched samples.

## Results

### Patient disposition and characteristics

**Figure 1:**
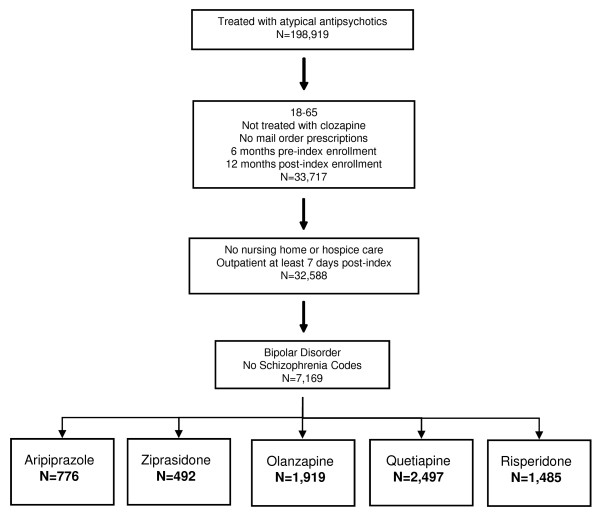
Patient flow

Of 198,919 patients with at least one atypical antipsychotic prescription, 7,169 met full inclusion criteria (Figure 1). Of these, 776 patients were on aripiprazole, 492 on ziprasidone, 1,919 on olanzapine, 2,497 on quetiapine and 1,485 on risperidone. Propensity score-matching enabled matching of: 461 aripiprazole and ziprasidone patients; 737 aripiprazole and olanzapine patients; 770 aripiprazole and quetiapine patients; and 771 aripiprazole and risperidone patients. Baseline characteristics after matching are shown in Table 1, demonstrating that comparable baseline characteristics were seen across all propensity score-matched treatment groups.

**Table 1:** Baseline and pre-index characteristics of propensity score-matched study sample

Variable	Aripiprazole (n = 461)	Ziprasidone (n = 461)	p-value	Aripiprazole (n = 737)	Olanzapine (n = 737)	p-value	Aripiprazole (n = 770)	Quetiapine (n = 770)	p-value	Aripiprazole (n = 771)	Risperidone (n = 771)	p-value
Age, mean (SD)	37.4 (11.6)	37.9 (11.1)	0.514	37.5 (12.0)	37.7 (11.9)	0.758	37.1 (11.9)	36.5 (11.4)	0.315	37.1 (11.9)	37.1 (11.2)	0.998
Sex, n (% men)	337 (73.1)	333 (722)	0.995	483 (65.5)	467 (63.4)	0.384	515 (66.9)	541 (70.3)	0.154	515 (66.8)	511 (66.3)	0.829
Psychiatric hospitalization, n (%)	159 (34.5)	160 (34.7)	0.945	179 (24.3)	182 (24.7)	0.856	178 (23.1)	178 (23.1)	1.000	180 (23.3)	180 (23.3)	1.000
Diabetes, n (%)	36 (7.8)	33 (7.2)	0.707	40 (5.4)	47 (6.4)	0.439	45 (5.8)	38 (4.9)	0.430	45 (5.8)	44 (5.7)	0.913
Hyperlipidemia, n (%)	75 (16.3)	81 (17.6)	0.598	123 (16.7)	131 (17.8)	0.581	130 (16.9)	130 (16.9)	1.000	130 (16.9)	119 (15.4)	0.446
Mood stabilizer exposure, n (%):												
Carbamazapine	13 (2.8)	15 (3.3)	0.701	25 (3.4)	27 (3.7)	0.778	28 (3.6)	29 (3.8)	0.893	27 (3.5)	29 (3.8)	0.893
Lamotrigine	72 (15.6)	75 (16.3)	0.787	116 (15.7)	115 (15.6)	0.943	137 (17.8)	134 (17.4)	0.841	136 (17.6)	134 (17.4)	0.841
Lithium	67 (14.5)	70 (15.2)	0.781	106 (14.4)	104 (14.1)	0.882	115 (14.9)	108 (14.0)	0.612	115 (14.9)	108 (14.0)	0.612
Oxcarbazepine	34 (7.4)	38 (8.2)	0.623	60 (8.1)	65 (8.8)	0.640	75 (9.7)	68 (8.8)	0.539	75 (9.7)	68 (8.8)	0.539
Topiramate	43 (9.3)	46 (10.0)	0.738	68 (9.2)	65 (8.8)	0.785	81 (10.5)	85 (11.0)	0.742	80 (10.4)	85 (11.0)	0.742
Valproate	83 (18.0)	84 (18.2)	0.932	159 (21.6)	156 (21.2)	0.849	165 (21.4)	168 (21.8)	0.853	165 (21.4)	168 (21.8)	0.853
Charlson comorbidity index, mean (SD)	0.3 (0.7)	0.4 (0.9)	0.388	0.3 (0.7)	0.3 (0.8)	0.432	0.2 (0.6)	0.2 (0.7)	0.746	0.3 (0.8)	0.3 (0.8)	0.468

P-values were calculated based on t-tests for continuous variables and chi square tests for categorical variables.

### Clinical outcomes

Table 2 describes the disposition and dosing for patients in each treatment group. Hospitalization rates among patients treated with aripiprazole ranged from 6.2 to 7.4% depending on the matched cohort, whereas comparators ranged from 9.3 to 12.8%. More than two-thirds of all patients discontinued their index antipsychotic during the 1-year follow-up period, and less than 5% completed a full year of follow-up taking their index antipsychotic medication. The duration of therapy on atypical antipsychotics was comparable across all treatment groups and fairly brief, with a median of 30 days across all treatments. Starting and maximal doses were relatively similar, suggesting limited titration after initiation.

**Table 2:** Patient disposition and dosing - study sample

		Psychiatric Hospitalization	Medical Hospitalization	Add/Switch Antipsychotic	Discontinued Antipsychotic	Completed Follow-up	Duration of Antipsychotic Treatment	Starting Daily Dose	Maximum Daily Dose
Index Antipsychotic	N	N (%)	N (%)	N (%)	N (%)	N (%)	Median days (Q1, Q3)	Mean mg (SD)	Mean mg (SD)
Aripiprazole	461	35 (7.6)	8 (1.7)	28 (6.1)	379 (82.2)	11 (2.4)	30 (30, 71)	11.8 (6.7)	13.4 (8.5)
Ziprasidone	461	59 (12.8)	11 (2.4)	66 (14.3)	307 (66.6)	18 (3.9)	30 (30, 70)	83.2 (49.7)	95.5 (57.2)
Aripiprazole	737	47 (6.4)	11 (1.5)	48 (6.5)	609 (82.6)	22 (3.0)	30 (30, 72)	11.2 (6.5)	12.9 (8.1)
Olanzapine	737	66 (9.0)	14 (1.9)	37 (5.0)	603 (81.8)	17 (2.3)	30 (30, 63)	7.8 (5.4)	8.7 (5.8)
Aripiprazole	770	48 (6.2)	10 (1.3)	49 (6.4)	640 (83.1)	23 (3.0)	30 (30, 72)	11.2 (6.5)	12.8 (8.1)
Quetiapine	770	78 (10.1)	8 (1.0)	34 (4.4)	619 (80.4)	31 (4.0)	30 (30, 73)	140.3 (146.1)	172.2 (200.6)
Aripiprazole	771	49 (6.4)	11 (1.4)	49 (6.4)	639 (82.9)	23 (3.0)	30 (30, 71)	12.8 (8.1)	12.8 (8.1)
Risperidone	771	72 (9.3)	14 (1.8)	59 (7.7)	603 (78.2)	23 (3.0)	30 (30, 73)	1.6 (1.3)	1.6 (1.3)

Fully adjusted Cox proportional hazards analysis demonstrated that treatment with aripiprazole was associated with a significantly lower risk of hospitalization than ziprasidone, olanzapine and quetiapine, and not significantly different than risperidone. Table 3 summarizes the results of these models, in which pre-index psychiatric hospitalization was significantly associated with risk of post-index hospitalization in all models. The number of pre-index mood stabilizers was not significantly associated with risk of hospitalization. Gender and age were not associated with risk of hospitalization in any cohort. There was variability among matched cohorts regarding the association between post-index mood stabilizer exposure and risk of hospitalization. Results of the analysis in unmatched samples are in Table 4. The effects are directionally the same, statistically significant, with some effect sizes being even larger than in the matched analyses.

**Table 3:** Adjusted Cox proportionate hazards models (aripiprazole reference)

Effect	Ziprasidone Hazard Ratio (95% CI)	Olanzapine Hazard Ratio (95% CI)	Quetiapine Hazard Ratio (95% CI)	Risperidone Hazard Ratio (95% CI)
Age	0.992 (0.973-1.011)	0.997 (0.981-1.014)	0.994 (0.978-1.011)	0.988 (0.971-1.005)
Women vs. Men	1.164 (0.720-1.882)	0.755 (0.510-1.118)	1.269 (0.837-1.924)	0.776 (0.524-1.149)
Charlson Comorbidity Index	1.220 (1.024-1.454)*	1.054 (0.876-1.267)	0.801 (0.548-1.171)	1.109 (0.958-1.284)
Prior Psychiatric Hospitalization	2.910 (1.888-4.484)***	3.541(2.408-5.207)***	3.874 (2.703-5.553)***	2.287 (1.579-3.314)***
Prior Diabetes	0.838 (0.338-2.076)	0.885 (0.358-2.189)	2.222 (0.943-5.232)	1.149 (0.550-2.400)
Prior Hyperlipidemia	0.913 (0.446-1.317)	0.614 (0.324-1.165)	0.895 (0.508-1.579)	1.327(0.776-2.268)
Prior Lipid Test	0.677 (0.348-1.317)	0.715 (0.412-1.243)	1.307 (0.779-2.194)	0.687(0.397-1.189)
Prior Glucose Test	1.172 (0.742-1.849)	1.409 (0.928-2.141)	0.792 (0.519-1.210)	1.476 (0.969-2.248)
Pre-index mood stabilizer				
1 vs. none	1.177 (0.648-2.138)	1.194 (0.679-2.098)	1.489 (0.904-2.451)	1.197(0.697-2.056)
2 vs. none	0.765 (0.270-2.171)	0.689 (0.280-1.691)	1.487 (0.704-3.142)	1.250(0.552-2.831)
≥ 3 vs. none	0.331 (0.035-3.137)	1.068 (0.291-0.919)	0.617 (0.073-5.217)	0.301(0.035-2.567)
Post-index mood stabilizer				
Carbamazepine	3.853 (1.623-9.151)**	2.191(0.988-4.859)	1.242 (0.579-2.661)	1.926(0.879-4.219)
Lamotrigine	1.268 (0.640-2.514)	1.233 (0.649-2.344)	0.844 (0.486-1.465)	1.482(0.853-2.573)
Lithium	0.716 (0.317-1.619)	2.287(1.249-4.188)**	0.710 (0.377-1.339)	1.029(0.556-1.904)
Oxcarbazepine	1.660 (0.740-3.726)	1.266 (0.607-2.640)	0.469 (0.201-1.092)	0.877(0.422-1.824)
Topiramate	1.256 (0.536-2.943)	1.699 (0.847-3.407)	0.800 (0.422-1.518)	1.362(0.697-2.663)
Valproate	0.811 (0.399-1.652)	0.754 (0.402-0.414)	0.438 (0.235-0.814)**	0.679(0.364-1.266)
Year of Index Prescription	0.963 (0.733-1.266)	1.047 (0.814-0.345)	0.773 (0.617-0.969)	0.758(0.604-0.952)*
Comparator vs. Aripiprazole	1.962 (1.269-3.033)**	1.554 (1.035-1.333)*	1.556 (1.078-2.245)*	1.368(0.940-1.989)

* p < 0.05

**p < 0.01

***p < 0.001

**Table 4:** Adjusted Cox proportionate hazards models (aripiprazole reference) for unmatched samples

Effect	Ziprasidone Hazard Ratio (95% CI)	Olanzapine Hazard Ratio (95% CI)	Quetiapine Hazard Ratio (95% CI)	Risperidone Hazard Ratio (95% CI)
Age	0.988 (0.971-1.006)	0.992 (0.980-1.004)	0.991 (0.980-1.001)	0.994 (0.981-1.007)
Women vs. Men	1.137 (0.735-1.759)	1.013 (0.773-1.326)	1.193 (0.928-1.533)	0.909 (0.675-1.224)
Charlson Comorbidity Index	1.168 (1.027-1.328)	1.054 (0.912-1.218)	0.988 (0.855-1.142)	1.100 (0.968-1.249)
Prior Psychiatric Hospitalization	2.805 (1.902-4.136)	3.051 (2.333-3.990)	2.777 (2.213-3.485)	2.551 (1.923-3.385)
Prior Diabetes	0.901 (0.414-1.965)	1.056 (0.550-2.026)	1.170 (0.704-1.944)	1.294 (0.738-2.269)
Prior Hyperlipidemia	1.255 (0.685-2.299)	0.834 (0.539-1.289)	0.828 (0.572-1.199)	1.162 (0.764-1.769)
Prior Lipid Test	0.641 (0.357-1.149)	0.807 (0.546-1.192)	1.000 (0.722-1.386)	1.048 (0.696-1.579)
Prior Glucose Test	1.305 (0.858-1.983)	1.288 (0.963-1.722)	1.030 (0.801-1.325)	1.132 (0.819-1.563)
Pre-index mood stabilizer				
1 vs. none	1.624 (0.946-2.789)	0.735 (0.499-1.085)	0.849 (0.606-1.188)	1.310 (0.863-1.988)
2 vs. none	1.241 (0.533-2.890)	0.387 (0.196-0.764)	0.646 (0.372-1.122)	1.737 (0.925-3.263)
≥3 vs. none	0.833 (0.162-4.287)	0.434 (0.134-1.408)	0.705 (0.225-2.207)	0.304 (0.038-2.416)
Post-index mood stabilizer				
Carbamazepine	2.792 (1.292-6.031)	2.741 (1.417-5.301)	1.745 (0.986-3.087)	1.482 (0.720-3.049)
Lamotrigine	1.204 (0.671-2.161)	1.765 (1.056-2.952)	1.058 (0.719-1.555)	1.273 (0.810-2.002)
Lithium	0.588 (0.298-1.159)	2.314 (1.509-3.551)	0.931 (0.626-1.385)	0.740 (0.452-1.210)
Oxcarbazepine	1.342 (0.655-2.748)	1.876 (1.034-3.403)	0.870 (0.519-1.459)	1.016 (0.584-1.766)
Topiramate	1.006 (0.493-2.051)	1.753 (0.994-3.091)	1.336 (0.844-2.117)	1.052 (0.596-1.855)
Valproate	0.646 (0.341-1.224)	1.352 (0.889-2.056)	0.824 (0.561-1.211)	0.782 (0.504-1.214)
Year of Index Prescription	0.937 (0.732-1.199)	1.032 (0.868-1.228)	0.963 (0.837-1.109)	0.959 (0.803-1.146)
Comparator vs. Aripiprazole	2.047 (1.388-3.019)	1.549 (1.098-2.184)	1.551 (1.139-2.113)	1.567 (1.124-2.186)

### Economic outcomes

Monthly post-index healthcare cost estimates derived from the gamma regression are summarized in Table 5. Adjusted monthly inpatient/ER mental health costs were significantly lower in the aripiprazole-treated patients compared with those treated with ziprasidone, olanzapine and quetiapine, and numerically lower than risperidone in those patients with inpatient costs. Total mental health costs were lower for aripiprazole compared to ziprasidone and quetiapine, but not significantly different compared to olanzapine and risperidone. Compared to aripiprazole, total medical costs were higher for quetiapine but not significantly different for all other comparators. Pharmacy costs were lower for olanzapine, risperidone and quetiapine, and not significantly different for ziprasidone. Total healthcare costs in the follow-up period were significantly lower for aripiprazole than quetiapine, and not significantly different for the other comparators. Results of the analysis in unmatched samples are in Table 6. The effects are directionally the same.

**Table 5:** Adjusted monthly post-index costs for patients with positive costs, US dollars

Cost Category	Aripiprazole Mean $ (SE)	Ziprasidone Mean $ (SE)	p-value	Aripiprazole Mean $ (SE)	Olanzapine Mean $ (SE)	p-value	Aripiprazole Mean $ (SE)	Quetiapine Mean $ (SE)	p-value	Aripiprazole Mean $ (SE)	Risperidone Mean $ (SE)	p-value
**Psychiatric costs**												
Inpatient/ER	788.70(91.60)	1039.90 (121.70)	0.076	666.90 (61.80)	876.20 (91.70)	0.038	627.60 (56.80)	833.80 (81.00)	0.024	674.40 (61.70)	743.60 (73.50)	0.446
Outpatient	202.00 (12.50)	271.90 (16.40)	<0.001	191.60 (9.70)	207.00 (9.90)	0.210	194.40 (9.20)	232.30 (10.90)	0.003	195.10 (9.50)	206.30 (9.50)	0.351
Total	487.20 (33.80)	631.20 (43.60)	0.004	447.30 (24.80)	483.70 (27.50)	0.287	429.90 (22.60)	518.80 (28.00)	0.007	449.10 (24.10)	441.50 (23.70)	0.807
**General medical costs**												
Inpatient/ER	747.20 (115.10)	686.40 (104.30)	0.687	681.00 (86.80)	372.20 (44.50)	<0.001	642.30 (83.70)	790.70 (89.70)	0.220	667.60 (87.40)	966.80 (120.30)	0.038
Outpatient	398.00 (24.60)	365.20 (23.30)	0.282	372.90 (19.30)	382.80 (20.00)	0.690	386.40 (19.60)	433.80 (21.20)	0.070	384.10 (19.20)	353.60 (17.30)	0.189
Total	540.20 (36.50)	527.10 (36.40)	0.777	521.60 (28.40)	484.60 (26.50)	0.294	519.40 (28.40)	655.70 (34.00)	0.001	511.10 (27.70)	542.10 (29.20)	0.395
**Psychiatric Medical and General Medical costs**	961.30 (59.00)	1055.10 (65.90)	0.223	910.00 (45.00)	891.20 (43.80)	0.736	875.00 (42.20)	1,060.30 (50.00)	0.001	898.20 (43.90)	934.30 (45.00)	0.518
**Pharmacy costs**	286.00 (11.10)	296.10 (11.40)	0.435	281.80 (9.10)	257.20 (8.00)	0.012	288.60 (9.00)	252.80 (7.70)	<0.001	282.70 (8.90)	241.00 (7.20)	<0.001
**TOTAL COSTS**	1,308.20 (64.90)	1,406.20 (70.80)	0.229	1,287.40 (51.10)	1,214.00 (47.70)	0.224	1,230.70 (47.70)	1,354.90 (51.70)	0.040	1,252.40 (49.60)	1,216.20 (47.60)	0.540

Results of generalized gamma regression adjusting for pre-index costs in propensity score-matched cohorts

**Table 6:** Adjusted monthly post-index costs for unmatched patients with positive costs, US dollars

Cost Category	Aripiprazole Mean $ (SE)	Ziprasidone Mean $ (SE)	p-value	Aripiprazole Mean $ (SE)	Olanzapine Mean $ (SE)	p-value	Aripiprazole Mean $ (SE)	Quetiapine Mean $ (SE)	p-value	Aripiprazole Mean $ (SE)	Risperidone Mean $ (SE)	p-value
**Psychiatric Medical costs**												
Inpatient/ER	678.00(61.10)	1025.70 (117.60)	0.003	660.70 (57..90)	931.10 (63.60)	0.001	665.50 (58.40)	859.40 (46.80)	0.010	667.00 (58.00)	786.50 (54.90)	0.121
Outpatient	189.30 (9.20)	267.80 (15.30)	<0.001	185.70 (8.80)	208.30 (6.70)	0.024	200.20 (8.80)	235.30 (6.50)	0.001	196.40 (9.00)	218.10 (7.40)	0.041
Total	450.30 (23.60)	639.70 (43.60)	<0.001	428.40 (22.70)	474.50 (18.50)	0.095	445.90 (22.70)	536.20 (17.40)	0.001	445.30 (22.90)	471.30 (18.80)	0.347
**General Medical costs**												
Inpatient/ER	648.10 (83.30)	752.00 (376.10)	0.431	635.60 (78.00)	488.90 (36.60)	0.063	665.60 (82.70)	821.60 (50.70)	0.124	664.40 (86.50)	854.80 (75.10)	0.103
Outpatient	373.90 (18.40)	376.10 (23.30)	0.931	387.40 (18.70)	366.80 (12.20)	0.312	386.90 (18.20)	434.10 (12.60)	0.025	373.10 (18.20)	363.60 (13.70)	0.646
Total	505.50 (27.00)	556.80 (36.40)	0.210	538.10 (27.30)	502.10 (17.50)	0.227	546.90 (27.90)	681.80 (21.20)	<0.001	491.70 (25.80)	518.80 (21.40)	0.377
**Psychiatric Medical and General Medical costs**	887.20 (42.00)	1,066.00 (64.40)	0.007	903.80 (41.80)	895.70 (29.50)	0.861	931.00 (41.90)	1,129.40 (33.00)	<0.001	883.90 (40.90)	944.80 (34.40)	0.205
**Pharmacy costs**	286.90 (9.00)	293.80 (10.40)	0.533	288.00 (8.70)	270.60 (5.40)	0.043	296.60 (8.70)	267.00 (5.10)	<0.001	284.30 (8.30)	240.80 (5.30)	<0.001
**TOTAL COSTS**	1,253.30 (47.50)	1,419.20 (67.80)	0.018	1,275.80 (47.10)	1,202.60 (31.70)	0.145	1,306.00 (47.50)	1,439.70 (34.80)	0.013	1,239.70 (46.50)	1,220.60 (36.50)	0.708

Results of generalized gamma regression adjusting for pre-index costs in unmatched cohorts

## Discussion

This study extends findings of a previous short-term retrospective cohort study that reported reduced risk of hospitalization and lower psychiatric treatment costs of patients with bipolar disorder treated with mood stabilizer and adjunctive aripiprazole compared to adjunctive ziprasidone, olanzapine and quetiapine during a 90-day follow-up period [13,14]. In this 1-year follow-up study, risk of hospitalization was lower in patients treated with aripiprazole with or without mood stabilizer compared to ziprasidone, olanzapine and quetiapine. Duration of therapy on atypical antipsychotic therapy was comparable across all atypical antipsychotics in this study, although the duration was brief relative to the follow-up period, lasting less than 3 months in 75% of cases. However, treatment guidelines recommend regimen simplification after patients are stabilized [7,11]. Therefore, in our sample, it is possible that the short duration of atypical antipsychotic therapy reflects stabilization of patients that allowed discontinuation of the atypical antipsychotic. Gianfrancesco et al. found somewhat longer treatment durations in a study of commercially insured patients treated with antipsychotics, with treatment durations of 7-10 months [19]. However, they allowed a gap of up to 120 days before ending a treatment episode, whereas our threshold of 15 days was much more conservative. To allow for meaningful comparative analysis of the cost data, intent-to-treat analysis was conducted for the cost analysis and patients were followed up for 1 year after their initial atypical antipsychotics treatment.

Antipsychotic doses observed also tended to be lower than label-recommended doses and demonstrated little titration over the course of treatment. These observations are consistent with other reports on atypical antipsychotic dosing in bipolar disorder [20,21]. Although we are not able to determine the reasons for these dosing patterns, it is possible that, due to concerns regarding tolerability or safety, physicians were reluctant to start patients on higher doses.

Along with the lower risk of psychiatric hospitalizations associated with aripiprazole compared to three of the four comparators, patients who initiated aripiprazole had lower psychiatric inpatient costs. These results suggest that treatment with aripiprazole tends to provide a valuable cost-offset in saving from decreased hospitalization risk and associated inpatient costs. In particular, the lower risk of hospitalization combined with lower total costs compared to quetiapine represent two attractive outcomes for formulary decision-makers responsible for the entire costs of care [22].

Observational studies can provide important insights into the outcomes of clinical practice in real-world settings, where dosing, titration and concomitant medications are not constrained by trial protocols. Such studies evaluate the effectiveness of treatments as they are actually used rather than when optimally dosed. We included the full range of observed dosing in our analysis based on the assumption that, in selecting medications, physicians also use what they believe is the most appropriate dosing and titration for that medication.

This study has several limitations. As a non-randomized retrospective study of observational data, it is possible that despite the use of propensity score matching and multivariate modeling, unobserved treatment selection bias may confound the results. Propensity score matching, however, is a widely accepted method for minimizing the effects of treatment selection bias in observational data [15]. Other approaches such as instrumental variables and Heckman's sample selection bias method may also be used in such settings [23,24], although the potential for residual confounding remains with all such methods. The consistency of our results in propensity score-matched and unmatched samples suggests that these findings are robust. However, the dataset we analyzed consists of patients from a single commercial health plan; results may not be applicable to chronic populations that are more likely to be covered by public sector insurance. Replication in other observational datasets is necessary to validate the robustness of these results.

Additionally, by restricting the analysis to an inception cohort, we were only able to study the effects of the initial choice of medication following an antipsychotic-free period and are thus limited to conclusions on initial antipsychotic selection rather than the effectiveness of a given medication under all circumstances. Based on our results, aripiprazole appears to be the most effective initial choice among atypical antipsychotics for the acute treatment of bipolar disorder, and these effects appear to persist in the post-acute phase. Finally, the study only followed patients until their first psychiatric hospitalization and did not address outcomes following adding, switching, or discontinuing antipsychotics, which may be common in this population. The analysis of such complex treatment patterns within claims data may be subject to high levels of unobservable confounding and difficult to interpret with respect to the contribution of individual medications across complex regimens. Specifically, it may be challenging to account for residual effects of prior medications following a switch, which is why we chose inception cohort design. Moreover, the reasons for adding versus switching antipsychotics would require detailed clinical information not available in this dataset to adjust for treatment selection bias. Therefore, our results are limited to outcomes only while the patient is on their initial antipsychotic medication for that episode of treatment.

## Conclusions

In adults with bipolar disorder, treatment with aripiprazole was associated with a lower risk of hospitalization than ziprasidone, olanzapine and quetiapine, and lower mental health costs than ziprasidone and quetiapine in the year following initial prescription. Total healthcare costs of patients treated with aripiprazole were lower than those treated with quetiapine.

## Competing interests

Edward Kim MD, MBA, Min You, MS, and Yonghua Jing, PhD, are employees of Bristol-Myers Squibb. Andrei Pikalov, MD, PhD, and Quynh Van-Tran, PharmD, are employees of Otsuka America Pharmaceutical, Inc.

## Authors' contributions

All authors contributed to the design and coordination of the study, statistical analysis of results and manuscript preparation.

## Pre-publication history

The pre-publication history for this paper can be accessed here:

http://www.biomedcentral.com/1471-244X/11/6/prepub
